# Pharmacologic profiling of patient-derived xenograft models of primary treatment-naïve triple-negative breast cancer

**DOI:** 10.1038/s41598-020-74882-4

**Published:** 2020-10-21

**Authors:** Reid T. Powell, Abena Redwood, Xuan Liu, Lei Guo, Shirong Cai, Xinhui Zhou, Yizheng Tu, Xiaomei Zhang, Yuan Qi, Yan Jiang, Gloria Echeverria, Ningping Feng, XiaoYan Ma, Virginia Giuliani, Joseph R. Marszalek, Timothy P. Heffernan, Christopher P. Vellano, Jason B. White, Clifford Stephan, Peter J. Davies, Stacy Moulder, W. Fraser Symmans, Jeffrey T. Chang, Helen Piwnica-Worms

**Affiliations:** 1 Center for Translational Cancer Research, Texas A&M University, Houston, TX USA; 2 Department of Experimental Radiation Oncology, The University of Texas MD Anderson Cancer Center, Houston, TX USA; 3 Department of Integrative Biology and Pharmacology, UT Health Science Center, Houston, TX USA; 4 Department of Bioinformatics and Computational Biology, The University of Texas MD Anderson Cancer Center, Houston, TX USA; 5 TRACTION Platform, The University of Texas MD Anderson Cancer Center, Houston, TX USA; 6 Department of Breast Medical Oncology, The University of Texas MD Anderson Cancer Center, Houston, TX USA; 7 Department of Pathology, The University of Texas MD Anderson Cancer Center, Houston, TX USA

**Keywords:** High-throughput screening, Screening, Breast cancer

## Abstract

Triple-negative breast cancer (TNBC) accounts for 15–20% of breast cancer cases in the United States, lacks targeted therapeutic options, and is associated with a 40–80% risk of recurrence. Thus, identifying actionable targets in treatment-naïve and chemoresistant TNBC is a critical unmet medical need. To address this need, we performed high-throughput drug viability screens on human tumor cells isolated from 16 patient-derived xenograft models of treatment-naïve primary TNBC. The models span a range of TNBC subtypes and exhibit a diverse set of putative driver mutations, thus providing a unique patient-derived, molecularly annotated pharmacologic resource that is reflective of TNBC. We identified therapeutically actionable targets including kinesin spindle protein (KSP). The KSP inhibitor targets the mitotic spindle through mechanisms independent of microtubule stability and showed efficacy in models that were resistant to microtubule inhibitors used as part of the current standard of care for TNBC. We also observed subtype selectivity of Prima-1^Met^, which showed higher levels of efficacy in the mesenchymal subtype. Coupling pharmacologic data with genomic and transcriptomic information, we showed that Prima-1^Met^ activity was independent of its canonical target, mutant p53, and was better associated with glutathione metabolism, providing an alternate molecularly defined biomarker for this drug.

## Introduction

Triple-negative breast cancers (TNBC) are known for their heterogeneous response to standard chemotherapy, with approximately 50% of newly diagnosed tumors having incomplete response to chemotherapy in the neoadjuvant setting. TNBCs lack hormone receptor and HER2 targets, precluding use of some of the most effective targeted treatments^1,2^. Notably, 30–40% of patients with localized disease that are treated with curative intent will have substantial residual cancer following neoadjuvant chemotherapy—and these patients have a very poor prognosis with a 40–80% risk of recurrence and death within 2–3 years of diagnosis^3–5^. Despite major advances in targeted cancer therapeutics, chemotherapy consisting of various combinations of DNA-damaging agents (anthracyclines, phosphoramide mustards, and platinum salts) and mitotic inhibitors (taxanes) remain the most commonly used agents for primary TNBC^4,6,7^. In patients with advanced disease, therapeutic resistance develops in virtually all cases and typically does so within only a few months.

High-throughput screening (HTS) is a powerful tool for repositioning established drugs towards novel clinical implications and for finding treatments that overcome drug resistance. However, the prognostic value of HTS is limited by the fidelity of the model systems being used, which should recapitulate critical aspects of the disease being studied. Historically, the most heavily utilized models in HTS have been established cell lines^8,9^, which may fail to recapitulate the behavior of primary cells and lack the heterogeneity seen in human tumors^10–12^. For these reasons, many translational studies now rely on patient-derived xenograft (PDX) models, which are thought to more accurately recapitulate the genomic heterogeneity of the patient tumors from which they were derived^11,13–16^. PDX models have been used to predict chemoresistance and uncover genetic mutations associated with drug activity^13,15,16^. In this study, we used a panel of orthotopic xenograft models generated from patients with newly diagnosed primary TNBC, who had not yet received neoadjuvant chemotherapy (NACT), as a resource for identifying targeted therapies with preclinical activity in primary TNBC.

## Results

### Overview of the study

**Figure 1:**
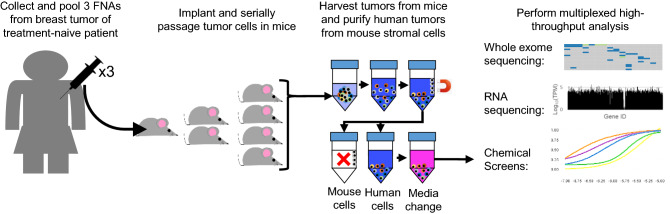
Schematic of experimental workflow. Diagram illustrating tumor collection from patient, serial propagation of patient tumor in mice, separation of patient-derived tumor cells from mouse stromal cells, and example of read-outs of whole exome sequencing, RNA-sequencing, and high-throughput chemical screens.

PDX models of treatment-naïve TNBC were established in alignment with an institutional review board-approved clinical trial (NCT02276443, Stacy Moulder, PI) at The University of Texas MD Anderson Cancer Center^17^. Tumor cells obtained by three fine-needle aspiration cores were pooled and engrafted into the fourth mammary fat pads of non-obese diabetic/severe combined immunodeficient (NOD/SCID) mice, and PDX lines were considered established after three consecutive passages in mice. When tumors reached ~ 1000 mm^3^, they were collected and tumor pieces were snap frozen in liquid nitrogen for whole exome sequencing, placed in RNAlater for RNA sequencing, and dissociated into single cell suspensions for orthotopic engraftment into new cohorts of mice. For drug screens, tumors were collected when they reached 1000 mm^3^ and digested into single cell suspensions, and mouse cells were depleted using an antibody-based magnetic purification system as previously described^18,19^. PDX-derived tumor cells (PDTCs) were then cultured in vitro for drug screening (Fig. 1).

### Molecular attributes of the PDX cohort

**Figure 2:**
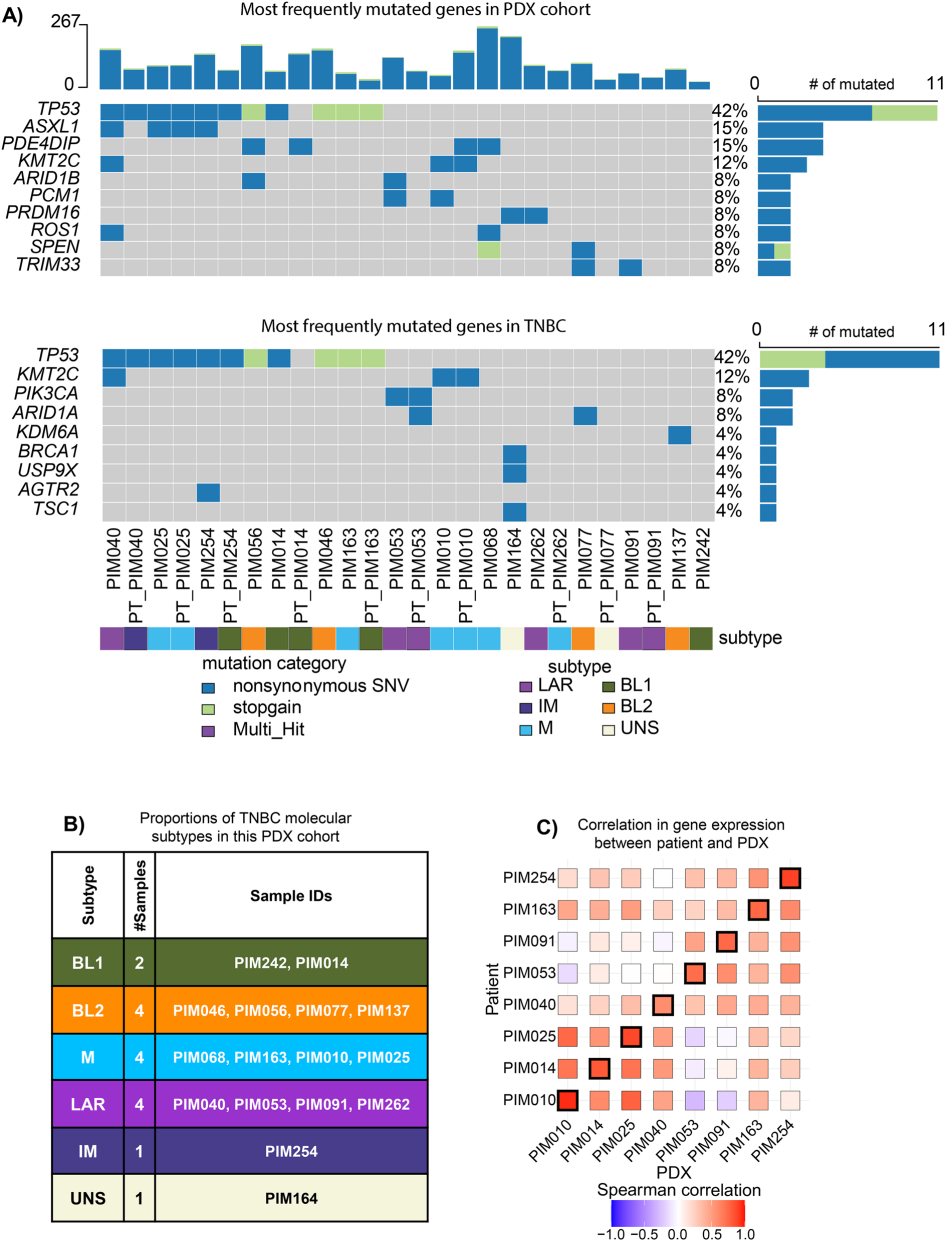
Characterization of PDX models. (**A**) Oncoplot of the most frequently mutated genes found in our PDX cohort and also found in TNBC^22^. Genomic samples from patients are denoted with a *PT_* prefix prepended to their respective PDX surrogate. (**B**) Molecular subtypes determined from PDX gene expression profile: BL1, basal-like 1; BL2, basal-like 2; IM, immunomodulatory; LAR, luminal androgen receptor; M, mesenchymal; UNS, unstable. (**C**) Correlative analysis of the 1000 most variant gene transcripts from RNA sequencing between patient tumors and PDX model.

We compared the genomes and transcriptomes of PDTCs from each PDX model to their respective patient tumor cells and to TNBC as a whole to establish the translational relevance of our models. In TNBC, *TP53* was the most frequently mutated gene. *TP53* mutation occurs in approximately 75% of cases, with more infrequent mutations occurring in other cancer-associated genes^20,21^. *TP53* mutation occurred in 47% of our PDX models (42% of samples when including patient data) with less frequent mutations found in other genes, consistent with what has been previously described in TNBC^22^ (Fig. 2A). We next applied the molecular sub-classification of TNBC^23^ to our PDX collection, which revealed that our cohort consisted of two basal-like 1 (BL1), four basal-like 2 (BL2), one immune-modulatory (IM), four luminal androgen receptor (LAR), four mesenchymal (M), and one unstable (UNS) subtypes (Fig. 2B), thus providing a diverse sampling of molecular subtypes that captured all but mesenchymal stem-like. To determine whether the gene expression profiles of the patient tumor cells were retained in their corresponding PDX tumor, we correlated the profiles of each PDX tumor sample with each patient tumor sample. The analysis showed a strong positive Pearson correlation coefficient between each model and its respective patient tumor (Fig. 2C). Taken together, these data demonstrate that our collection of PDX models maintain characteristics of their respective patient tumors and are representative of the majority of TNBC subtypes from both mutational and transcriptomic perspectives. Finally, we looked at the sub-clonal architecture of the nine matched patient and PDX derived models with high quality samples. While we cannot rule out undetected large-scale clonal expansion events, the exome sequencing revealed smaller shifts in the prevalence of subclones of approximately 30% (Supplemental Fig. 1). Similarly, we observed that certain mutations were lost when comparing the patient and PDX samples (Fig. 2A). While this is a potential limitation in the translational relevance of these models, others have shown that the retention of the exact sub-clonal architecture is not necessary to recapitulate pharmacologic susceptibilities *in vivo*^24,25^.

### Generation of chemical profiles

**Figure 3:**
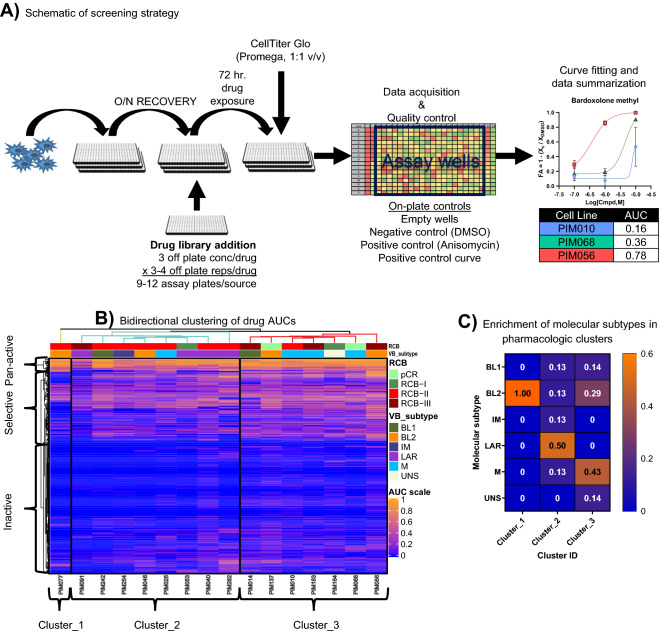
Generation of high-throughput chemical profiles. (**A**) Schematic representation of the experimental procedures for high-throughput chemical screening assays. (**B**) Bi-directional hierarchical clustering of AUC values generated from the three-point dose–response curves. Color scale = AUC values ranging from 0 (Inactive) to 1 (Strong active), RCB, residual cancer burden, VB subtype, Vanderbilt molecular classification generated from PDX RNA-seq data. (**C**) Enrichment of subtypes in pharmacological clusters denoted by the color or branches.

Next, we generated chemical profiles for PDTCs from each PDX model using the method schematized in Fig. 3A. In brief, PDTCs were seeded into 384-well plates and grown as non-adherent suspensions in MammoCult medium (Supplemental Fig. 2A) and allowed to recover overnight. Next, the PDTCs were screened against a mechanistically annotated chemical library composed from the Broad Informer Library (CTRP_vr2), the NCI-approved clinical oncology library (version 5), and an in-house library of clinical oncology drugs totaling 634 chemical entities with a drug exposure window of 72 h, which was previously optimized using growth data (Supplemental Fig. 2B). We demonstrated a highly consistent Z′, a common measure of assay robustness, with an average performance greater than 0.75 across all PDX models. Further, we showed a high degree of technical reproducibility with an average minimum significance ratio below two, indicative of a highly robust and technically reproducible assay (Supplemental Fig. 3A,B). Primary screening assays were done with 3–4 technical replicates at three dose concentrations (10.0, 1.0, 0.1 uM), depending on the number of viable PDTCs isolated, which varied between PDX models. Drug effects were normalized to the vehicle control (DMSO 0.1% v/v) using the following formula *FA* = *1-(X*_*c*_*/X*_*DMSO*_*),* where X_c_ is the luminescence signal for each data point and X_DMSO_ is the median luminescence from on-plate DMSO control wells. The normalized data were then fit across the three-point dose–response curve and summarized by calculating the area under the fitted curve. One potential limitation of this method is that it fails to account for alterations in tumor cell growth rate that may confound chemical susceptibilities^26^. Another limitation of this data set is the limited resolution provided by a fixed-concentration three-point dose–response curve, which may fail to recapitulate chemical selectivity for very potent drugs. However, quantification of sub-maximal efficacy is still possible in those contexts. Despite these limitations, we were still able to draw a series of relevant conclusions from the drug screening results obtained using the methods outlined above.

Hierarchical clustering of the pharmacologic profiles across PDX models demonstrated that LAR and M subtypes tended to co-cluster (Chi-squared *p* = 0.37, *p* = 0.19 when cluster_1 was excluded as an orphan), while the basal-like subtypes were intermixed (Fig. 3B,C). Next, we used chemical target and pathway annotations to visualize the response of PDTCs to families of drugs used in the screen. Analysis of AUC data for compounds targeting the same pathway or with similar mechanism of action showed highly correlated response profiles (Supplementary Fig. 3C). These results are another metric attesting to the reproducibility and validity of the drug screeing results. Independent clustering of chemical entities revealed three sub-clusters, which we subsequently refer to as “inactive”, “selective”, and “pan-active” (Fig. 3B). Inactive describes those drugs that showed no or minimal activity in the majority of PDTCs, whereas pan-active describes drugs that exhibited broad activity across many models. Members of the anthracycline family (e.g., doxorubicin, epirubicin, and duanorubucin) were pan-active in these screens, further validating the use of these drugs as a front-line therapy in TNBC. Selective describes drugs showing large differences in susceptibility across models, and these can provide insight into therapeutically actionable targets that are relevant to specific molecular subtypes or can describe heterogeneity in molecular signaling cascades; thus, selective drugs are interesting from a personalized medicine standpoint.

### Inhibition of mitotic spindle proteins

**Figure 4:**
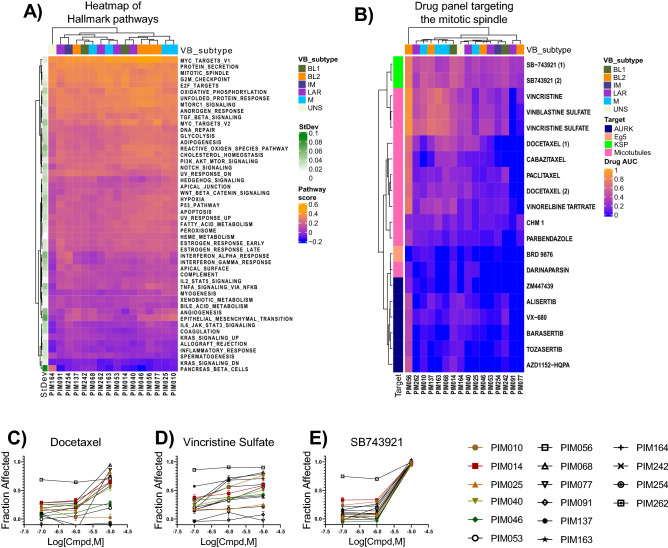
Targeting the mitotic spindle. (**A**) Heatmap showing the Z-normalized hallmark pathways across PDX models. Row side bar reflects the standard deviation of raw scores. (**B**) Bi-directional hierarchical clustering of AUC values for drugs that target the mitotic spindle through multiple mechanisms. Color scale = 0 (Inactive) to 1 (Strong active), VB subtype, Vanderbilt molecular classification purposed by Lehmann et al. (2011). BL1, basal-like 1; BL2, basal-like 2; IM, immunomodulatory; LAR, luminal androgen receptor; M, mesenchymal; UNS, unstable. (**C**–**E**) Dose–response curves of a prototypic taxane (docetaxel), vinca alkaloid (vincristine sulfate), and KSP inhibitor (SB-743921), respectively.

We first looked at molecularly defined pathway activity from single sample gene set enrichment analysis (ssGSEA) to provide a starting point for rationalizing the observed drug activities. From this analysis, we selected the Hallmark pathway representations, which are a well-recognized and curated set of 50 pathways^27^. The mitotic spindle pathway was ranked as one of the most consistently active pathways across all PDTCs (Fig. 4A). Indeed, indirect targeting of the mitotic spindle using microtubule poisons such as paclitaxel is considered the mainstay neoadjuvant chemotherapy for TNBC^28^. Yet multiple PDTCs tested here exhibited resistance to taxane-based therapies, which was a feature that was recapitulated in matched PDX models treated in vivo with paclitaxel (Supplemental Fig. 4). Given the clinical importance of this drug and the observation of resistance in treatment-naïve TNBC, we sought to identify alternate compounds that could inhibit the mitotic spindle with improved activity.

The panel of drugs that target the mitotic spindle in this library include agents that target microtubule stabilization (e.g., taxanes), microtubule destabilization (e.g., vinca alkaloids), microtubule associated kinesin proteins (e.g., kinesin spindle protein [KSP] inhibitors), and mitotic kinases (e.g., AURKA inhibitors) (Fig. 4B)*.* From the HTS data, we observed that taxanes had a broad spectrum of activity, including activity against PDTCs that were partially or fully resistant to paclitaxel in vitro (Fig. 4C). Vincristine sulfate and related molecules showed greater potency relative to taxanes; however, complete resistance was still observed in some cases (Fig. 4D). Thus, despite the potency of taxanes and vinca alkaloids, we observed sub- maximal efficacy in certain PDTCs, which reflect the presence of resistant clonal populations within these tumors. Further studies are warranted to identify mechanisms of resistance. However, the KSP inhibitor SB-743921, which was present in two of the sub-libraries, was pan-active and fully efficacious in all PDTCs tested and reduced the viability of PDTCs that were resistant to both taxane and vinca in vitro (Fig. 4D,E).

### Subtype selective drug activity

**Figure 5:**
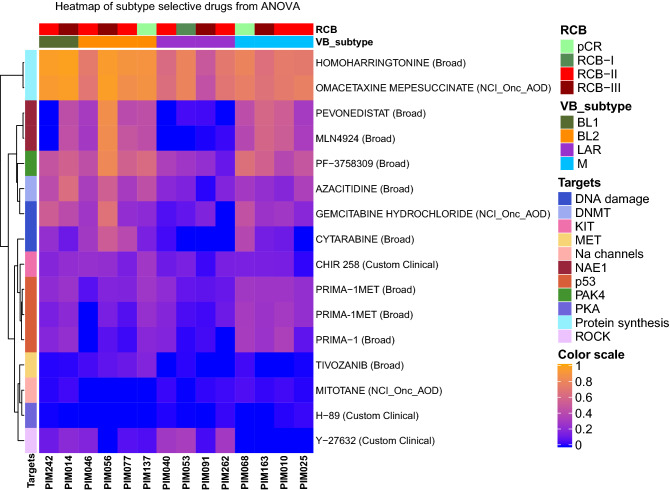
Subtype-selective molecules. Bi-directional hierarchical clustering of AUC values for drugs with a significant interaction (ANOVA *p* < 0.05) across molecular subtypes.

Having observed the trend that LAR and M subtypes were separable using pharmacologic profiles (Fig. 3B,C), we next investigated the selectivity of targeted agents across molecular subtypes. To address this, drug activity was compared across molecular subtypes, excluding the IM and UNS classifications due to limited observations, using analysis of variance (ANOVA). From this analysis, we identified 16 drugs that had at least one significant interaction (*p* < 0.05) between molecular classes (Fig. 5). Protein synthesis inhibitors (homoharringtonine and omacetaxine mepesuccinate) and inhibitors of Kit/FLT3 (Chir 258) and VEGFR/MET (tivozanib) showed moderately higher levels of sensitivity in basal-like subtypes. LAR was characterized with significant loss of sensitivity to multiple drugs targeting NAE1 (pevonedistat and MLN4924), DNA-damaging agents (gemcitabine hydrochloride and cytarabine), and PAK4 inhibitors (PF-3758309) in addition to the targets mentioned above. Interestingly, the ROCK inhibitor Y-27632 displayed selectivity in LAR PDX models. PDX models from the M subtype had higher activity with Prima-1 and Prima-1^Met^.

### Exploration of Prima-1^Met^ selectivity

**Figure 6:**
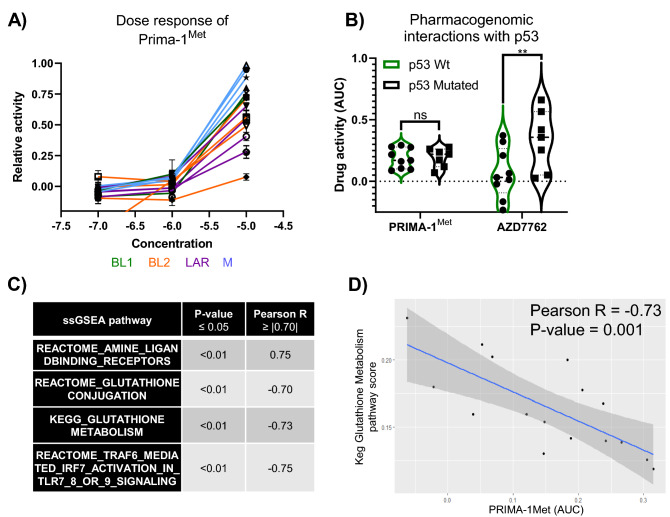
Prima-1^Met^ pharmaco-genomic and transcriptomic associations. (**A**) Dose–response curve from primary screen. (**B**) Violin plot of AUC values vs *TP53* status for Prima-1^Met^ (*p* > 0.999) and AZD7762 (*p* < 0.01). (**C**) List of ssGSEA pathways that had a significant (*p* < 0.05) and strong correlation (absolute value of Pearson r coefficient ≥ 0.70) with the AUC of Prima-1^Met^. (**D**) Example of the correlation between Prima-1^Met^ to KEGG glutathione metabolism pathway activity.

Prima-1 and its analogue Prima-1^Met^ (also known as APR-246) showed higher efficacy in the M subtype, with appreciable but lower efficacy in BL2 backgrounds (Fig. 6A). The primary mechanism of action for these drugs is to covalently modify mutant p53, resulting in a conformational change that restores activity^29^. Because *TP53* is the most frequently mutated gene in this cohort and in TNBC as a whole, we investigated whether there was a pharmacogenomic association between Prima-1^Met^ activity and *TP53* mutation status. ANOVA and post-hoc Sidak multiple testing correction revealed no significant interaction between Prima-1^Met^ sensitivity and *TP53* mutation status (*p* > 0.99). However, this analysis did identify a significant pharmacogenomic interaction between Chk1 inhibitor AZD7762 and *TP53* mutation (*p* < 0.01), which confirms the findings of other research^30^ and provides additional validation of this method (Fig. 6B). Overexpression of MDM2 can also phenocopy *TP53* loss of function mutations, which prompted us to explore whether there was an association between the AUC of Prima-1^Met^ activity and the gene expression of *MDM2*, which showed no significant correlation (Pearson *p* = 0.88, r = 0.05). Collectively, these data led us to explore potential alternate mechanisms to rationalize Prima-1^Met^ activity.

Prima-1^Met^ has been shown to induce apoptosis by increasing the expression of *TP73* and *NOXA* and depleting glutathione pools, resulting in increases in reactive oxygen species, independent of *TP53* mutation status^31^. In an unbiased correlative analysis between drug activity and ssGSEA pathways, we found significant correlations with glutathione metabolism and conjugation (Fig. 6C,D). These data are consistent with established alternate mechanisms of action for Prima-1^Met^ and implicate Prima-1^Met^ as working through a glutathione-dependent mechanism in this context.

## Conclusions and clinical implications

In this study, we established and validated a preclinical pharmaco-genomic resource generated from PDX models from representative treatment-naïve tumors of patients with primary TNBC. We performed multiplexed high-dimensional analysis including bulk whole exome and RNA sequencing as well as high-throughput chemical screening of human tumor cells to generate a resource for pharmaco-genomic and transcriptomic studies aimed at understanding and targeting TNBC. This resource expands upon other breast cancer-focused PDX biobanks by providing a molecularly diverse, TNBC-specific collection of models with matched pharmacologic profiles^12,16^. In addition, the PDX models in our collection were derived from the primary breast tumors of untreated TNBC patients. Most published PDX collections to date have been generated from primary or metastatic tumors from pretreated patients^16^. Thus, our collection provides a unique resource to study tumors that have not undergone therapy-induced selection and to identify potential therapies for targeting resistance in the setting of primary, treatment-naïve breast cancer.

Other high-throughput chemical screens with PDTCs have identified a wide range of actionable susceptibilities, which were largely recapitulated in our dataset. For example, Matossian et al*.* identified DNA synthesis, microtubule, and topoisomerase inhibitors as the top classes of drugs exhibiting activity against PDX-derived mesenchymal tumor cells^32^. In a study by Bruna et al*.*, anthracyclines, NAMPT and BCL-2 inhibitors were found to have the highest levels of activity against a subset of 10 basal-like TNBCs, while responses to taxanes and mTOR inhibitors were more variable^16^. We observed varying degrees of activity to DNA synthesis, microtubule, and mTOR inhibitors in our study, while anthracyclines, BCL-2 and NAMPT inhibitors tended to be pan-active. In a study by Turner et al., a panel of 10 short-term cultures of PDTCs were screened against ~ 1300 drugs at a single concentration of 10 µM using bioluminescence as the primary read-out of viability^33^. A panel of four basal-like TNBC PDX models were then prioritized and tested in combination with the proteasome inhibitor carfilzomib or the EGFR inhibitor afatinib against a library of 176 pan-active drugs identified in the primary screen. From this, the survivin inhibitor YM-155 demonstrated synergy when combined with afatinib. In our study, YM-155 was strongly active in all PDTCs while afatinib was moderately active, and carfilzomib was inactive in the majority of basal-like PDTCs. Another study testing a panel of 12 chemotherapeutic agents on basal-like PDTCs, demonstrated susceptibility towards bortezomib, dacarbazine, and cyclophosphamide^34^. In our study bortezomib was pan-active while dacarbazine and cyclophosphamide were inactive, albeit we tested those drugs at greater than 100-fold lower concentrations. Cross comparisons of all of these datasets should take into account drug concentrations, conditions used for culturing PDTCs, and therapies that the tumors had been exposed to prior to the PDX model generation.

Exploratory analysis of the pharmacologic profiles generated from our PDX cohort identified multiple targeted therapies with appreciable preclinical activity. Importantly, many of the drugs identified in our study have direct translational applicability, with pre-established pharmacokinetic and toxicity profiles already performed in other disease implications. These include targeted therapies to overcome intrinsic taxane resistance, which is considered a mainstay therapy in TNBC, using an alternate mechanism to target the mitotic spindle. Targeting mitotic proteins such as KSP has previously been proposed as an efficacious mechanism to target the mitotic spindle. Other research groups have observed potent inhibition of cell growth in immortalized cell lines and antineoplastic potential in xenograft models with KSP inhibitors^35^. KSP inhibitors were also identified as a potentially synergistic target when combined with vinblastine in TNBC using a computational approach that combined contrasting phenotypes^36^. A first-in-human phase I/II clinical trial using SB-743921 in advanced solid tumors showed efficacy and a more favorable toxicity profile compared to taxanes^37^. Our data, in combination with published literature, support revitalized interest in using KSP inhibitors, and further mechanistic studies in the context of TNBC are warranted.

Prima-1^Met^ demonstrated selectivity towards the M subtype, which was independent of *TP53* mutation status. A recently completed phase I clinical trial conducted in Sweden using single-agent Prima-1^Met^ in hematologic and prostate malignancies showed measurable target engagement (i.e., induction of genes regulated by p53) in vivo. As of 2019, phase II/III clinical trials have begun in the United States using Prima-1^Met^ in combination with azacitidine for myelodysplastic syndrome and acute myeloid leukemia^38^. Others have proposed using Prima-1^Met^ in TNBC due to the high rate of *TP53* mutations^39^. In those studies, Prima-1^Met^ was tested against a panel of TNBC cell lines with and without *TP53* mutations to demonstrate p53-dependent activity^39,40^. However, in our study we did not observe *TP53* mutation-dependent activity but rather found glutathione pathways, an established alternate target of Prima-1^Met^, as a potential alternate biomarker predictive of activity. Thus, use of Prima-1^Met^ has the potential to impact a large spectrum of TNBC patients who either harbor *TP53* mutations or have alterations in glutathione metabolism. Further validation of these findings will be required to translate them into clinical settings.

## Methods

### Collection of patient-derived materials

All research conducted in human patients followed national guidelines including the Common Rule (https://www.hhs.gov/ohrp/humansubjects/commonrule/) and the Health Insurance Portability and Accountability Act (HIPPA) privacy and security rules^41^. All patients from whom samples were collected for generation of PDXs gave informed consent and were enrolled in the ARTEMIS trial (NCT02276443) a MD Anderson IRB-approved protocol (2014-0185).

### Animals

All experimental procedures were approved by the Institutional Animal Care and Use Committee (IACUC) at MD Anderson Cancer Center under IACUC protocol 00000978-RN01. Endpoints for animal experiments were selected in accordance with IACUC-approved criteria. Female NOD/SCID mice (NOD.CB17-Prkdcscid/NcrCrl) were obtained from Charles River, National Cancer Institute Colony.

### PDX cell preparation for drug screen

The fourth mammary fat pads of 3- to 5-week-old female NOD/SCID mice were implanted with 50,000 PDX tumor cells suspended in 30 µL of DMEM/F12 (HyClone, Cat. No. SH30023.01) media mixed with Matrigel (Corning, Cat. No. 354234) (50:50). Cells in Matrigel were maintained on ice until engraftment. Tumors were monitored weekly. When tumors reached about 1000 mm^3^, they were harvested and dissociated into single cells and organoids by mechanical mincing, followed by digestion with collagenase (3 mg/mL; Roche) and hyaluronidase (0.6 mg/mL; Sigma-Aldrich) supplemented with 2% bovine serum albumin (Sigma-Aldrich) in DMEM/F12 containing antibiotics (penicillin (100 U/mL), streptomycin (100 µg/mL), and amphotericin B (0.25 µg/mL). Tumor digests were incubated on a rotating platform for 4 h at 37 °C. Digested PDX tumor cells were re-suspended in red blood cell lysis buffer (Sigma, Cat. No. R7757), then treated with 0.25% Trypsin-EDTA (Corning, Cat. No. MT25053CI) followed by 5 U/mL Dispase (Stemcell Technologies, Cat. No. 07913) and 1 mg/mL DNase I solution (Stemcell Technologies, Cat. No. 07900). Finally, cells went through magnetic-activated cell sorting using mouse cell depletion kits (Miltenyi Biotec: Mouse Cell Depletion Kit, 130-104-694) to remove mouse cells. On average, 40 million PDTCs were isolated and subjected to the drug screening process. For samples with residual material, we performed qPCR to determine the human to mouse DNA content in order to validate the purification process. This was done using a human RNaseP gene probe (20 × human RNaseP copy number assay, FAM-TAMRA, Life Technologies) and a mouse Trfc gene probe (20 × mouse Trfc copy number assay, VIC-TAMRA, Life Technologies). The relative ratio of human to mouse gDNA in each sample was calculated using the ΔΔCt method as described previously^19^. The human DNA content was determined to be 99.4% for PIM010 and 94.1% for PIM091 after mouse cell depletion.

### Administration of paclitaxel in vivo

Mice were randomly assigned to treatment or control arms and treatment was initiated when a palpable tumor was detected. Paclitaxel was formulated in Crem:Ethonal (1:1 v/v) and dosed at 15 mg/kg once a week by intraperitoneal injection. Methylcellulose (0.5%) was used as a vehicle control and dosed on the same schedule. Crem:Ethanol was made by adding 10 mL Kolliphor EL (Sigma, Cat No. C5135-500 g) to 10 mL ethanol (Pharmco-AAPER, USP grade, Cat: 64-17-5), followed by stirring for 3 h.

### Development and optimization of a high-throughput chemical screen

To optimize assay conditions for establishing short-term cultures of PDTCs (Supplemental Fig. 2A), we generated a single 384-well plate assay to simultaneously optimize cell seeding densities, test different culture media, and determine the growth properties of PDTCs under each condition. Different culture media were tested in the presence and absence of serum over three different cell seeding densities, and cell growth was monitored using an ATP bioluminescent reporter (CellTiter-Glo) over 7 days (Supplemental Fig. 2B). From these experiments, we determined that MammoCult medium maximally supported the viability and growth of PDTCs in vitro over 7 days in roughly half of the 24 models tested. In this medium, cells grew as either single-cell or self-associating non-adherent suspensions (Supplemental Fig. 4A). PDTCs that maintained viability or proliferative capacity were prioritized for full screening. Medium supplemented with serum improved viability of some PDTCs; however, serum-containing medium was not considered for screening assays due to the potential of depleting stem-like populations and altering tumor cell behavior. It was also determined that seeding 2000 cells/well was optimal for the screens. Once culture conditions were optimized, a focused screen was performed to identify a library of compounds that could be used as controls for tumor cell killing. This library consisted of eight non-redundant toxic oncology drugs (top four shown in Supplemental Fig. 2C). Anisomycin was found to robustly decrease cell viability across all PDX models and was used as an on-plate control to monitor assay performance during screening.

### Screening assays

Before plating, cell number and viability were determined by mixing 10 uL of culture media containing tumor cells with 10 uL trypan blue solution in a disposable counting slide, which was then read using a TC10 automated cell counter (Bio-Rab). Next, 2000 viable cells/well were transferred into barcoded 384-µ well clear plates (Griener, Cat No. 781091) using a MultiDrop Combi Reagent dispenser (Thermo). All drug libraries were diluted in DMSO and arrayed on Echo certified low dead volume plates (LDV, Labcyte). Drugs were subsequently transferred from the LDV source plate into assay plates using an Echo liquid handling machine (Labcyte). Wells were treated such that the final concentration of DMSO in media did not exceed 1% (v/v).

### Statistics and software

We processed the next-generation sequencing data using the BETSY system^42^. We called variants and estimated gene expression values as previously described^19^, except that we identified contaminating mouse host reads for subtraction using Xenome^43^. PyClone^44^ was used to identify sub-clonal populations in the patient and PDX data. Using this software, sub-clones were identified using a robustness score of greater than 0.5 and had to contain at least 2 mutations. Data organization and statistical analysis was performed using an automated screening tracking workflow developed in Pipeline Pilot (2018 Server, BIOVIA) and R statistics. The log concentration was fitted to the normalized response using a cascade model which leverages iteratively reweighted least squared to fit the response surface to a four-parameter logistic or linear model. AUC and IC_50_ values were generated from the fitted dose–response curve. Dose–response figures used for this publication were rendered in Graphpad Prism. Identification of subtype selective molecules was performed using the AOV function in R. Pharmacogenomic associations with mutant *TP53* and time series analysis of in vivo taxane data where performed and graphed using Graphpad Prism.

## Supplementary information

Supplementary Figures.

## Data Availability

Normalized data frames (PDTC by Drug AUC, Gene expression (TPM), and ssGSEA pathway scores) and open-source code that are critical to the analysis and generation of figures are available at https://github.com/ReidTPowell/TNBC_profiles_1. Analytical workflows generated in proprietary environments are available upon reasonable request.
