# Supplementary material for: Pharmacologic profiling of patient-derived xenograft models of primary treatment-naïve triple-negative breast cancer
Source: Sci Rep. 2020 Oct 21;10:17899. doi: 10.1038/s41598-020-74882-4 (PMC7578025; doi:10.1038/s41598-020-74882-4)
Supplement: Supplementary file 1 — Supplementary Figures. [file 41598_2020_74882_MOESM1_ESM.docx]

**­­­­­Pharmacologic profiling of patient-derived xenograft models**

**of primary treatment-naïve triple-negative breast cancer**

Reid T Powell^1^, Abena Redwood^2^, Xuan Liu^3^, Lei Guo^1^, Shirong Cai^2^, Xinhui Zhou^2^, Yizhseng Tu^2^, Xiaomei Zhang^2^, Yuan Qi^2,4^, Yan Jiang^2^, Gloria Echeverria^2^, Ningping Feng^5^, XiaoYan Ma^5^, Virginia Giuliani^5^, Joseph R. Marszalek^5^, Timothy P. Heffernan^5^, Christopher P. Vellano^5^,

Jason B. White^6^, Clifford Stephan^1^, Peter J. Davies^1^, Stacy Moulder^6^,

W. Fraser Symmans^7^, Jeffrey T. Chang^3,4,^ and Helen Piwnica-Worms^2*^

*1) Center for Translational Cancer Research, Texas A&M University, Houston, TX*

*2) Department of Experimental Radiation Oncology, The University of Texas MD Anderson Cancer Center, Houston, TX*

*3) Department of Integrative Biology and Pharmacology, UT Health Science Center, Houston, TX*

*4) Department of Bioinformatics and Computational Biology, The University of Texas MD Anderson Cancer Center*

*5) TRACTION Platform, The University of Texas MD Anderson Cancer Center, Houston, TX*

*6) Department of Breast Medical Oncology, The University of Texas MD Anderson Cancer Center, Houston, TX*

*7) Department of Pathology, The University of Texas MD Anderson Cancer Center, Houston, TX*

* Corresponding author: hpiwnica-worms@mdanderson.org

**
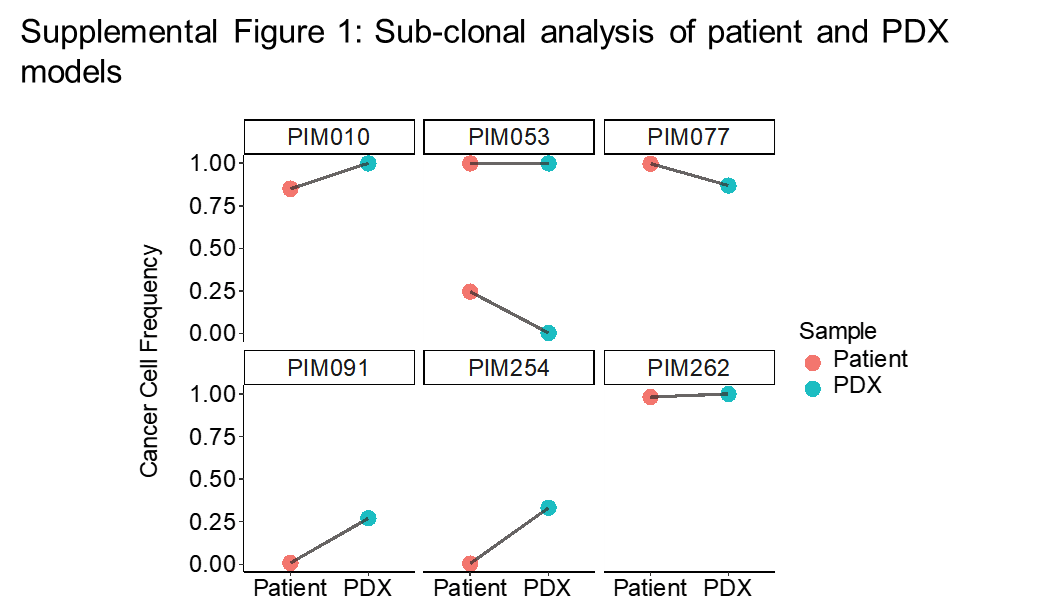
**

**Supplemental Figure 1: Sub-clonal analysis of patient and PDX models:** Analysis of clonal architecture was performed using PyClone. The lines show the cancer cell frequencies (y-axis) of somatic mutations found in clones or subclones in the patient or PDX samples.

**
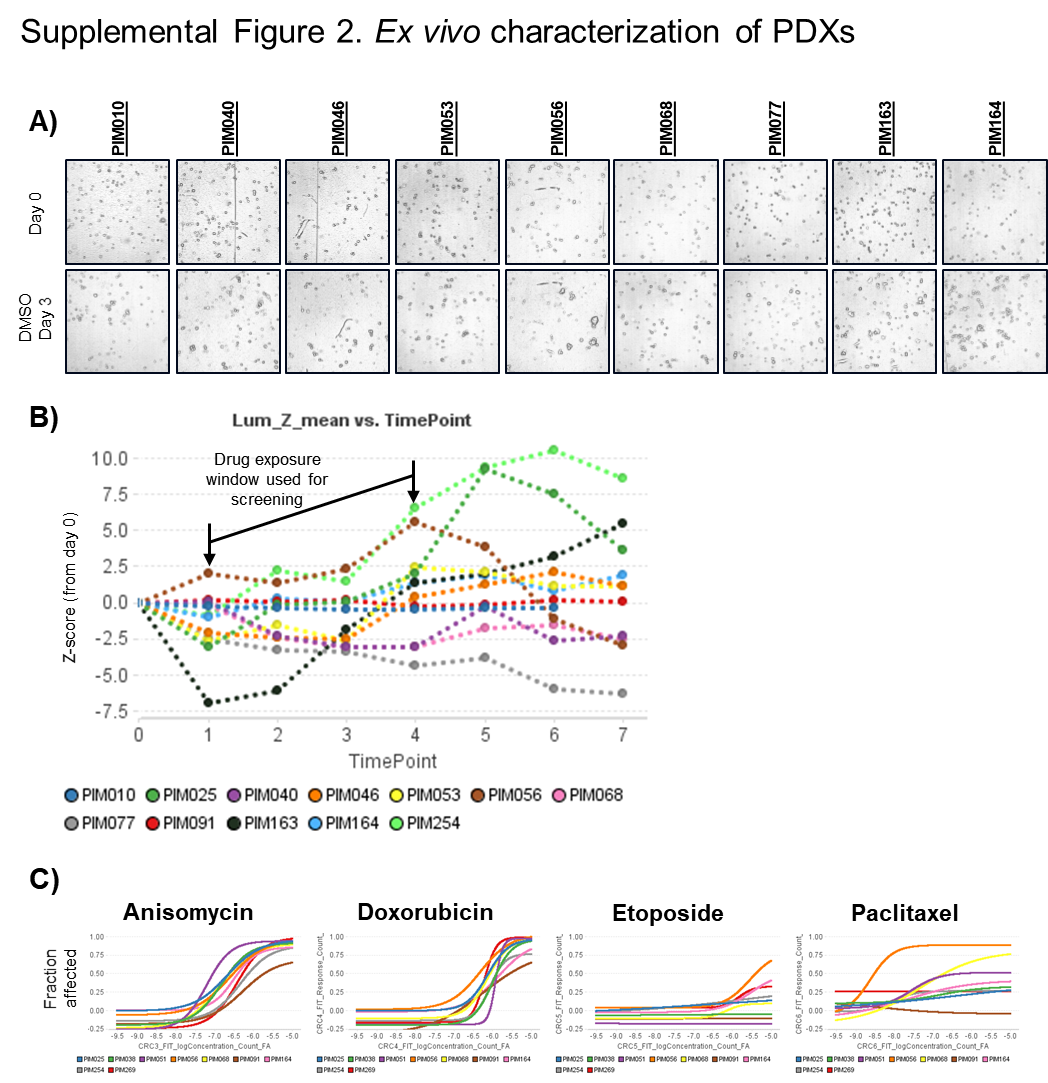
Supplemental Figure 2: Ex vivo characterization of PDXs:** A) Representative bright field images of tumor cell cultures at the day of plating and after 3 days of DMSO exposure. Images were acquired at 4x and cropped to a consistent scale for viewing. B) Growth of tumor cells measured by CellTiter glow luminescence over a 7 day period. C) Dose response curves of top 4 compounds used to identify a robust on-plate positive control.

*
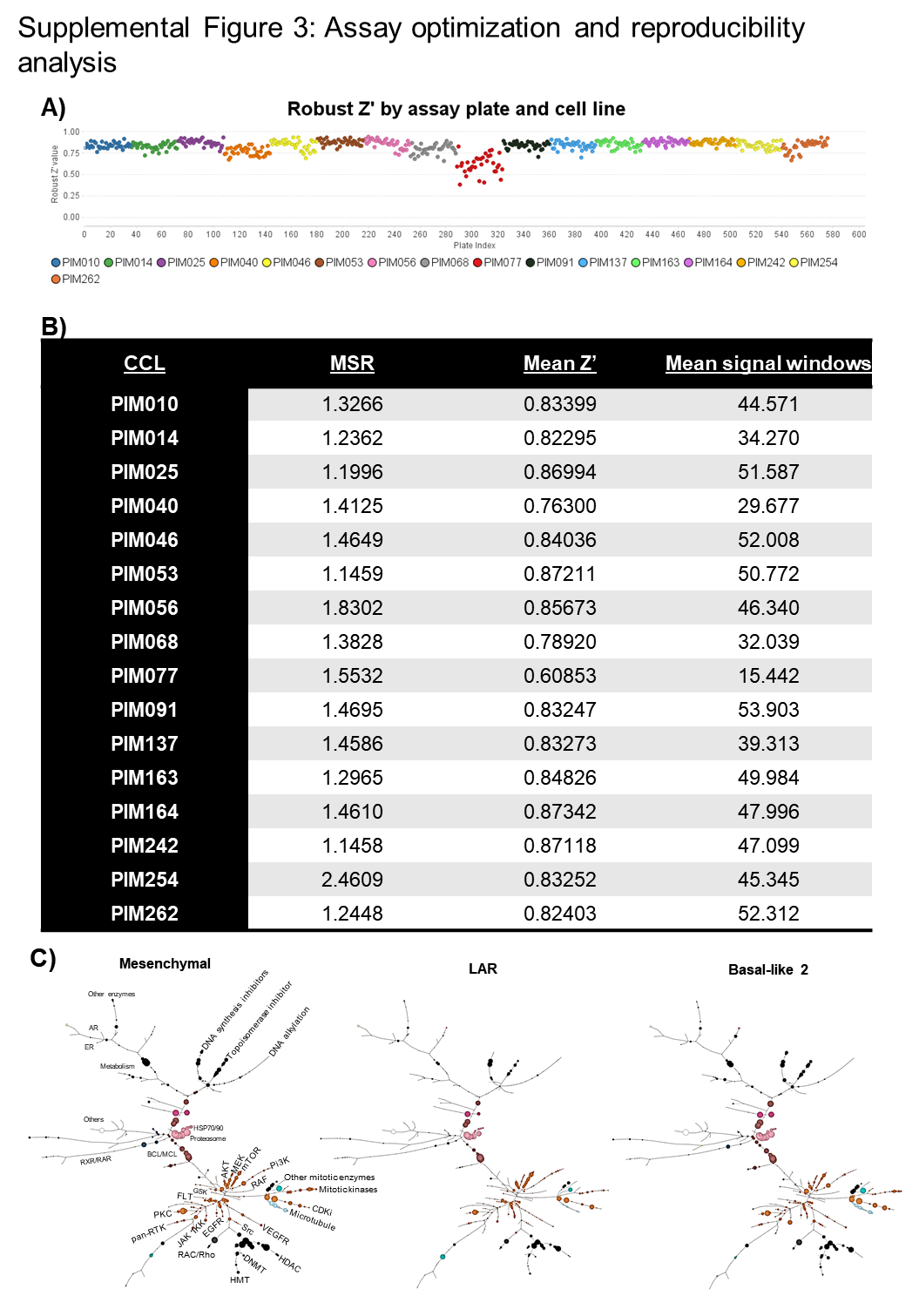
*

**Supplemental Figure 3: Assay optimization and reproducibility analysis:** A) Robust Z-prime (Z’) values calculated from on plate controls. Each data point represents an individual plate, colors are assigned by PDX model. B) Table of assay quality control metrics. Minimum significant ratio (MSR), Z-prime (Z’). C) In-house chemical ­­­similarity diagram generated from target and pathway annotations showing prototypic responses of Mesenchymal, LAR, and Basl-like-2 response profiles. Dots represent individual drugs, size is scaled to the AUC of drug acti­vity, families of drugs active against the same target are more proximal.

*
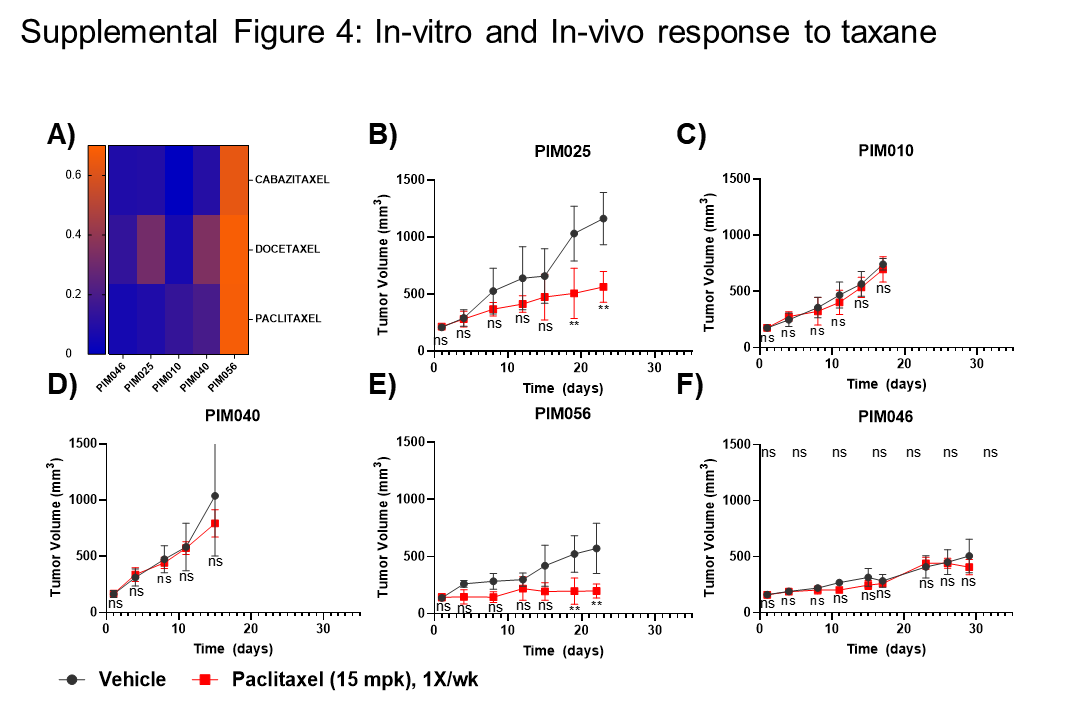
*

**Supplemental Figure 4: Response to taxane In vitro and in vivo.** A) Heatmap showing AUC values of taxanes from in vitro HTS that have corresponding in vivo data. B-F) Response of PDX models treated with 15 mpk paclitaxel or vehicle one time per week. Data-points and error bars represent the mean and standard deviation, respectively, of the tumor volume calculated from three mice. Statistical significance between the two cohorts over time was determined using a two-way ANOVA and post-hoc Sidak multiple comparisons test performed in Graphpad Prism. ns (p> 0.05), ** (p<.01)
